# Association between body mass index and fragility fracture in postmenopausal women: a cross-sectional study using Korean National Health and Nutrition Examination Survey 2008–2009 (KNHANES IV)

**DOI:** 10.1186/s12905-021-01209-4

**Published:** 2021-02-09

**Authors:** Jihan Kim, Sami Lee, Sung Soo Kim, Jong-Pyo Lee, Jong Sung Kim, Jin Gyu Jung, Seok Jun Yoon, Kyu Pil Kim, Chan-Keol Park, Yong-Hwan Kim

**Affiliations:** 1 Department of Family Medicine, Sejong Trinium Woman’s Hospital, Sejong, Korea; 2 Department of Family Medicine, Research Institute for Medical Science, Chungnam National University School of Medicine, 282 Munhwa-ro, Jung-Gu, Daejeon, 35015 Korea; 3 Department of Obstetrics and Gynecology, Sejong Trinium Woman’s Hospital, Sejong, Korea; 4 Department of Family Medicine, Chungnam National University Sejong Hospital, Sejong, Korea; 5 Division of Rheumatology, Department of Internal Medicine, Chungnam National University Sejong Hospital, Sejong, Korea; 6 Department of Orthopedic Surgery, Chungnam National University Hospital, Daejeon, Korea

**Keywords:** Body mass index, Obesity, Osteoporosis, Osteoporotic fractures, Postmenopausal

## Abstract

**Background:**

The present study examined the relationship between body mass index (BMI) and the risk for fragility fractures in postmenopausal Korean women.

**Methods:**

Among subjects who participated in the 4th Korea National Health and Nutrition Examination Survey (2008–2009), 2114 women ≥ 40 years of age were included. BMI was based on standards set by the Korean Society for the Study of Obesity, as follows: < 18.5 kg/m^2^, underweight; 18.5 ≤ to < 25 kg/m^2^, normal weight; and ≥ 25 kg/m^2^, obese. Subjects were also divided into three groups according to the location of fragility fracture: spine, hip, or wrist.

**Results:**

The mean (± SD) rate of fragility fracture was significantly different among the three groups: 5.9 ± 2.9% (underweight), 1.1 ± 0.3% (normal weight), and 3.0 ± 0.7% (obese) (*p* = 0.001). After correcting for age, family history, and treatment history of osteoporosis and rheumatoid arthritis, smoking and drinking status, and level of exercise, multivariable regression analysis revealed that the odds ratio for fragility fracture in the underweight group was 5.48 [95% confidence interval (CI) 1.80–16.73] and 3.33 (95% CI 1.61–6.87) in the obese group. After subdividing fragility fractures into vertebral and non-vertebral, the odds ratio for vertebral fracture in the underweight group was 5.49 (95% CI 1.31–23.09) times higher than that in the normal weight group; in the obese group, the non-vertebral fracture odds ratio was 3.87 (95% CI 1.45–10.33) times higher. Analysis of non-vertebral fractures in the obese group revealed an odds ratio for fracture 22.05 (95% CI 1.33–365.31) times higher for hip fracture and 3.85 (95% CI 1.35–10.93) times higher for wrist fracture.

**Conclusions:**

Obesity and underweight increased the risk for fragility fractures in postmenopausal Korean women.

## Background

Osteoporosis is a musculoskeletal disorder characterized by a decrease in bone density and abnormal changes in bone microstructure [1]. It is one of the most common diseases in postmenopausal women due to the age-related reduction in estrogen levels. Individuals with osteoporosis have a higher risk for fragility fractures due to reduced bone density, and increased risk for resulting complications and mortality rates [2]. Fragility fracture refers to a non-traumatic fracture and, according to the World Health Organization (WHO), is defined as a fracture caused by a fall from a level below one’s height, including fracture(s) caused by impact that do not cause fracture(s) in normal bones, and even when that impact is not recognized [3]. In Korea, women aged > 50 (59.5%) are more likely to experience fragility fractures in their lifetime than men > 50 (23.8%) years of age [4]. Therefore, in Korea, the National Health Examination Program, under the National Health Insurance Service, was designed for women aged 54 and 66 to undergo testing for bone mineral density in the lumbar spine to diagnose, treat, and manage diseases related to osteoporosis.

One of the most common changes experienced by postmenopausal women is weight gain. Compared with premenopausal women, postmenopausal women exhibit a 20% increase in fat mass, especially in central adiposity [5]. In general, it is common for postmenopausal women to exhibit a decrease in bone density with age; however, weight gain has been reported to help maintain bone density [6]. Therefore, weight loss was believed to increase the risk for fragility fracture due to a reduction in bone density while, conversely, an increase in body weight would maintain bone density and eventually prevent fragility fracture. However, in recent years, it has been considered important to not simply observe a change in weight but rather a change in muscle mass. Therefore, increase in body weight—not due to muscle mass, but to body fat—could increase the risk for osteoporosis [7]. In fact, studies including Korean women have demonstrated that high body fat mass can increase the risk for bone density reduction in the femoral neck [8]. In another study including postmenopausal women, the optimal body mass index (BMI) with minimal risk for osteoporosis was 23–24.9 kg/m^2^ [9]. As such, weight gain is no longer believed to prevent osteoporosis.

Although several studies have investigated the relationship between obesity and osteoporosis in Koreans, only a few have examined the impact of obesity on fragility fractures. Accordingly, this study aimed to investigate the relationship between BMI—an index of obesity—and fragility fractures in postmenopausal Korean women.

## Methods

### Participants

The Korean National Health and Nutrition Examination Survey is a statutory survey addressing the health behavior, prevalence of chronic disease, and food and nutrition intake of Korean by the Korea Centers for Disease Control and Prevention and is a government-designated statistic based on Article 17 of the Statistics Act (Approval No. 117002). It corresponds to research conducted by the government for public welfare in accordance with the Bioethics Act. The original survey data are freely accessible without any administrative permission as open-access resources through the website of the Korea Centers for Disease Control and Prevention (https://www.knhanes.cdc.go.kr) [10].

**Fig. 1:**
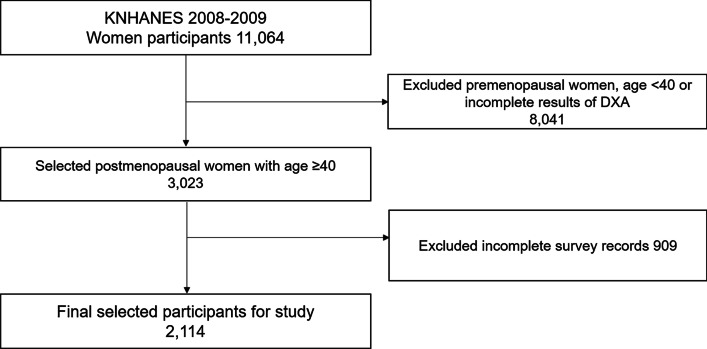
Study population. KNHANES, Korean National Health and Nutrition Examination Survey; DXA, Dual-energy X-ray absorptiometry

A total of 11,604 women registered in the 4th National Health and Nutrition Examination Survey (2008–2009) were selected as primary participant group. Of these, 3023 were postmenopausal, ≥ 40 years of age, and had available bone density test data, and were selected as the secondary participant group. After exclusion of 909 women with uncertain examination records, 2114 were ultimately included as the final participant group (Fig. 1).

### Definitions and measurements

BMI was determined by dividing body weight (kg) by height (m^2^). Based on the WHO standards for the Western Pacific region and Korean Society for the Study of Obesity standards, BMI < 18.5 kg/m^2^ was defined as underweight, 18.5 ≤ to < 25 kg/m^2^ as normal weight, and ≥ 25 kg/m^2^ as obese [11]. In the present study, those classified as overweight, with BMI 23 ≤ to < 25 kg/m^2^, were allocated to the normal weight group according to the National Health and Nutrition Examination Survey questionnaire.

Fragility fractures were defined as non-traumatic or low-traumatic fractures caused by the impact of a fall below one’s height [12]. For fragility fractures, responses to the question “Have you experienced fragility fractures?”, were divided into sites such as the “spine,” “hip,” and “wrist,” with responses “Yes,” “No,” “Not Applicable,” and “I don't know.” Those who responded “I don't know" were already excluded during the selection process. “Yes” refers to people diagnosed with osteoporosis or osteopenia through bone mineral density tests such as dual-energy X-ray absorptiometry (DXA) and experienced fractures. “No” refers to the group diagnosed with osteoporosis or osteopenia through tests, but didn’t experience fractures. “Not applicable” refers to the group who were not diagnosed with bone related disease through tests and did not experience fractures as well. Since it was necessary to determine the presence or absence of a fracture in this study, those who responded with “No” and “Not Applicable” were allocated to the same group with no fragility fractures. Participants who responded with “Yes” to any of the three sites were classified into a group with fragility fractures.

Bone density in the participants was measured using DXA and a fan-beam densitometer (Discovery, Hologic Inc., Bedford, MA, USA), and the lumbar spine and left femur were measured. The units of bone density were area density, g/cm^2^. In addition, according to the osteoporosis diagnostic criteria set by the WHO, those with T-score > − 1.0 were classified as normal, T-score > − 2.5 and ≤ − 1.0 as osteopenia, and T-score ≤ − 2.5 as osteoporosis [13].

In the smoking questionnaire, current smokers refer to those who have smoked 100 or more cigarettes in a lifetime and continue to smoke at the time of the survey. Former smokers refer to those who smoked 100 or more cigarettes in the past and have not continued to smoke at the time of the survey. Non-smokers refer to those who have never smoked at least 100 cigarettes in their lifetime [14]. For alcohol status, considering the guidelines for moderate drinking of Koreans, the frequency of drinking from the question “Drinking frequency in a year” and the amount from the question “Amount you drink at a time” were both considered. Those who do not drink at all were classified as “non-drinkers,” those ≥ 65 years of age and who consumed < 2 glasses per week, or < 65 years of age and consumed < 4 glasses per week as “moderate drinkers,” and the remainder as “heavy drinkers” [15]. The moderate exercise was classified based on the questionnaire about whether one exercises moderately for ≥ 30 min at least five times per week.

Waist circumference was measured to the nearest under 0.1 cm using SECA 200, a measuring tape, during whole exhalation at the horizontal plane midway between the lower end of the rib and the iliac crest, at the mid-axillary line [16].

### Statistical analysis

During the analysis of data from the National Health and Nutrition Examination Survey, the following errors were corrected through the complex sample design: inclusion errors due to differences in the number of households and populations between the sample design period and the survey period; unequal selection probabilities; and nonresponse error of those who did not participate in the survey. For this, the primary sampling unit, k-strata, and weight were specified [17].

Participants were divided into three groups according to BMI: underweight, normal weight, and obese. The complex samples general linear model was used for continuous variables such as age, height, waist circumference, age at menopause, and bone density (BMC, T-score) for mean comparison. For categorical variables, such as fragility fracture rate, treatment history and family history of osteoporosis, history of rheumatoid arthritis, smoking status, drinking status, and moderate exercise, Rao–Scott chi-square test was used.

Complex samples logistic regression was used to analyze the relationship between BMI and fragility fracture rate. During analysis, variables that could affect fragility fractures such as age, treatment history and family history of osteoporosis, history of rheumatoid arthritis, smoking status, drinking (i.e., alcohol) status, and moderate exercise, were corrected.

All statistical analyses in this study were performed using SPSS version 22 (IBM Corporation, Armonk, NY, USA).

## Results

### General characteristics of the participants

The mean (± SD) age of the normal weight group was 59.01 ± 0.42 years, which was not statistically different from the mean age of the underweight group (61.43 ± 3.00 years) but was significantly different from that of the obese group (60.98 ± 0.44 years) (*p* = 0.001). Among body composition items, mean height was not different among the three groups; however, weight, BMI, and waist circumference were different from the normal weight group. The mean weight of the normal, underweight, and obese groups was 53.62 ± 0.21 kg, 41.43 ± 1.05 kg, and 64.65 ± 0.28 kg, respectively; the differences were statistically significant (underweight, *p* < 0.001; obese, *p* < 0.001). The mean BMI of the normal, underweight, and obese groups was 22.49 ± 0.06 kg/m^2^, 17.55 ± 0.20 kg/m^2^, and 27.31 ± 0.09 kg/m^2^, respectively (underweight group, *p* < 0.001; obese group, *p* < 0.001). The mean waist circumferences of the normal, underweight, and obese groups were 76.96 ± 0.29 cm, 65.30 ± 0.86 cm, and 89.47 ± 0.37 cm, respectively, which was significantly different from the normal weight group (underweight group, *p* < 0.001; obese, *p* < 0.001). The average age at menopause was not different among the groups (normal weight, 48.59 ± 0.22 years; underweight, 45.61 ± 1.66 years; obese, 48.48 ± 0.26 years). The average weight-corrected appendicular skeletal muscle mass was 34.29 ± 0.37 for normal weight group, 49.43 ± 3.35 for underweight group, and 28.52 ± 0.33 for the obese group, with the underweight group and the obese group showing statistically significant difference compared to the normal weight group (each *p* < 0.001). In case of bone density (g/cm^2^), the average bone density of the femoral neck (0.79 ± 0.03) and the entire femur (0.94 ± 0.03) in the underweight group was significantly higher than that of the normal weight group (femoral neck average, 0.73 ± 0.01; femur average 0.88 ± 0.01) (each *p* = 0.035, *p* = 0.029) In the case of T-score, only the average T-score of the femoral neck in the underweight group (− 0.35 ± 0.24) was significantly different from that of the normal weight group (− 0.87 ± 0.06) (*p* = 0.032). Regarding fragility fracture rate, there were differences among the three groups: underweight, 5.9% ± 2.9; normal, 1.1 ± 0.3%; and obese, 3.0 ± 0.7% (*p* = 0.001). In the smoking status, there was a difference in the proportion of non-smoker, former smoker and current smoker in the three groups (*p* = 0.043, *χ*^2^ = 11.863). There were no statistical differences in treatment history and family history of osteoporosis, history of rheumatoid arthritis, drinking status, and moderate exercise among the three groups (Table 1).

**Table 1:** Characteristics for postmenopausal women according to BMI

	Underweight	Normal	Obesity	*p *value
	(BMI < 18.5)	(BMI ≥ 18.5 and < 25)	(BMI ≥ 25)	(χ^2^)
	N = 45	N = 1234	N = 835	
Est. number (*n*) (%)	105,855 (2.1%)	3,030,556 (60.6%)	1,867,253 (37.3%)	
Age (years)	61.43 ± 3.00	59.01 ± 0.42	60.98 ± 0.44^b^	
Height (cm)	153.36 ± 1.61	154.28 ± 0.22	153.77 ± 0.28	
Weight (kg)	41.43 ± 1.05^c^	53.62 ± 0.21	64.65 ± 0.28^c^	
BMI (kg/m^2^)	17.55 ± 0.20^c^	22.49 ± 0.06	27.31 ± 0.09^c^	
Waist circumference (cm)	65.30 ± 0.86^c^	76.96 ± 0.29	89.47 ± 0.37^c^	
Menopausal age (years)	45.61 ± 1.66	48.59 ± 0.22	48.48 ± 0.26	
Weight-adjusted ASM	49.43 ± 3.35^c^	34.29 ± 0.37	28.52 ± 0.33^c^	
BMD (g/cm^2^)				
Lumbar spine total	0.92 ± 0.03	0.90 ± 0.01	0.91 ± 0.01	
Femur neck	0.79 ± 0.03^a^	0.73 ± 0.01	0.72 ± 0.01	
Femur total	0.94 ± 0.03^a^	0.88 ± 0.01	0.88 ± 0.01	
BMD (T-score)				
Lumbar spine total	− 0.80 ± 0.22	− 0.91 ± 0.06	− 0.88 ± 0.06	
Femur neck	− 0.35 ± 0.24^a^	− 0.87 ± 0.06	− 0.89 ± 0.06	
Femur total	0.31 ± 0.18	− 0.03 ± 0.05	− 0.07 ± 0.06	
Fragility fracture (%)	5.9 (± 2.9)	1.1 (± 0.3)	3.0 (± 0.7)	0.001 (11.186)
Treatment of OP (%)				0.369
None	91.8 (± 3.6)	91.4 (± 1.0)	93.2 (± 1.1)	(1.938)
Current treatment	8.2 (± 3.6)	8.6 (± 1.0)	6.8 (± 1.1)	
FHx of osteoporosis (%)				0.540
No	78.3 (± 8.0)	85.1 (± 1.3)	86.0 (± 1.6)	(1.859)
Yes	21.7 (± 8.0)	14.9 (± 1.3)	14.0 (± 1.6)	
Rheumatoid arthritis (%)				0.156
No	94.0 (± 3.7)	96.3 (± 0.6)	94.3 (± 0.9)	(3.835)
Yes	6.0 (± 3.7)	3.7 (± 0.6)	5.7 (± 0.9)	
Smoking status (%)				0.043
Non-smoker	76.7 (± 7.8)	90.4 (± 1.0)	91.8 (± 1.1)	(11.863)
Former smoker	9.6 (± 5.0)	5.2 (± 0.7)	3.8 (± 0.7)	
Current smoker	13.7 (± 6.6)	4.4 (± 0.7)	4.4 (± 0.9)	
Alcohol consumption (%)				0.242
Non-drinker	65.4 (± 9.3)	52.4 (± 1.6)	49.1 (± 2.2)	(8.583)
Moderate drinker	23.9 (± 7.5)	40.4 (± 1.6)	44.6 (± 2.3)	
Heavy drinker	10.8 (± 7.6)	7.2 (± 0.9)	6.3 (± 1.1)	
Moderate exercise (%)				0.264
Yes	6.9 (± 4.1)	12.8 (± 1.3)	15.4 (± 1.9)	(3.855)
No	93.1 (± 4.1)	87.2 (± 1.3)	84.6 (± 1.9)	

All values are weighted value and presented mean (± standard deviation) or percent (± standard deviation)

a, b and c are presented *p* value < 0.05, < 0.01 and < 0.001, respectively

*BMI* body mass index, *ASM* appendicular skeletal muscle mass, *BMD* bone mineral density, *OP* osteoporosis, *FHx* familial history

### *Difference between vertebral and non-vertebral fracture rate according to BMI *via* Rao–Scott chi-squared test*

**Fig. 2:**
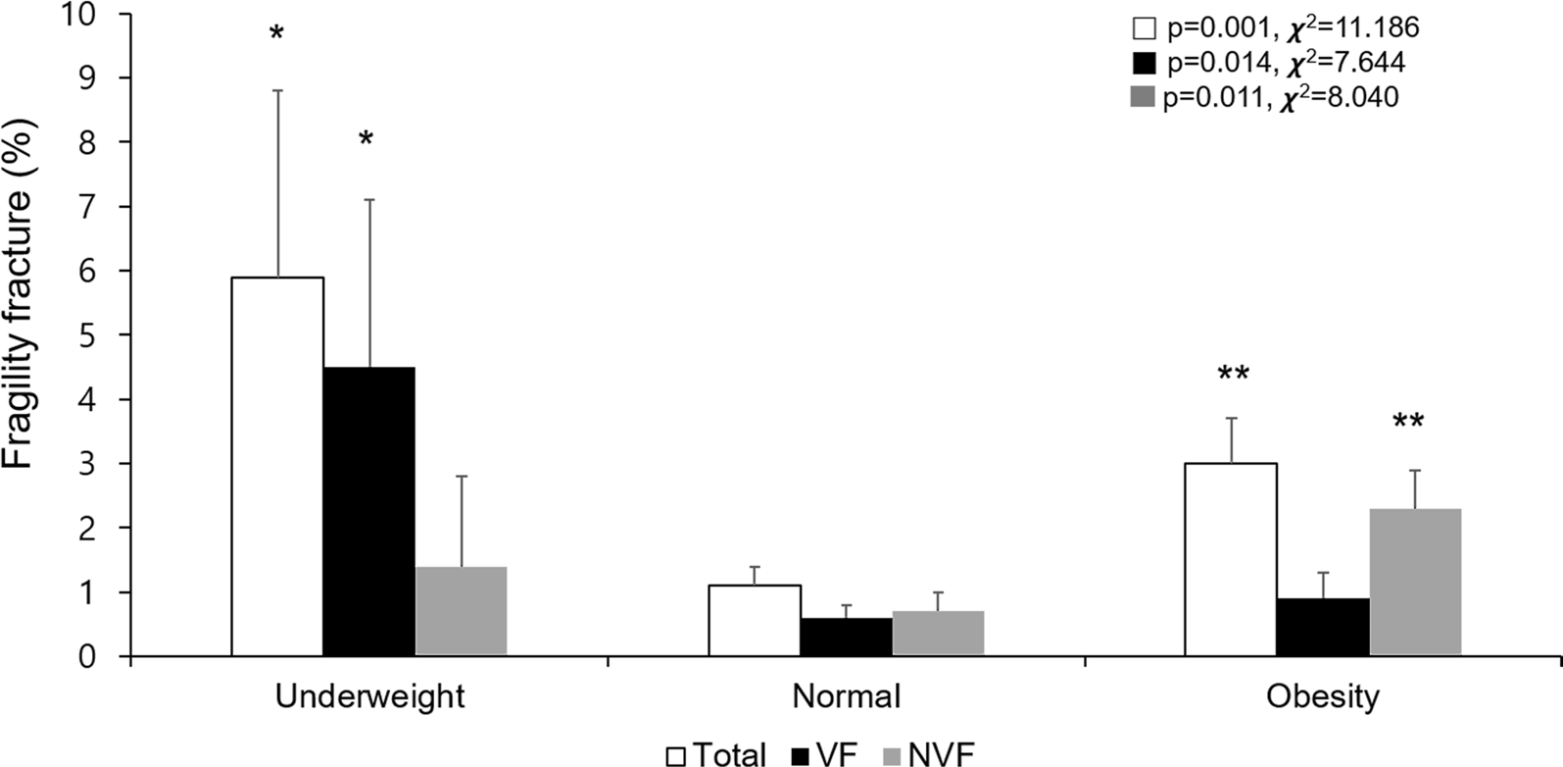
Prevalence of fragility fracture according to body mass index. * and ** means *p* < 0.05 and *p* < 0.01 compared to normal BMI by Rao–Scott chi-square test. *VF* vertebral fracture, *NVF* non-vertebral fracture, *BMI* body mass index

The overall fragility fracture rates for the underweight, normal weight, and obese groups were 5.9%, 1.1%, and 3.0%, respectively, and the difference was statistically significant (*p* = 0.001, χ^2^ = 11.186). When fracture rates were compared between the vertebral and non-vertebral fracture rates, vertebral fracture rate for the underweight group (4.5 ± 2.6%) was significantly higher compared with the normal weight group (0.6 ± 0.2%) and obese group (0.9 ± 0.4%) (*p* = 0.014, χ^2^ = 7.644). In the case of non-vertebral fracture rate, that of the obese group (2.3 ± 0.6%) was relatively higher than that of the normal weight group (0.7 ± 0.3%) and underweight group (1.4 ± 1.4%), with a statistically significant difference among the three groups (*p* = 0.011, χ^2^ = 8.040) (Fig. 2).

### Odds ratio for fragility fracture according to multiple logistic regression analysis

The odds ratio for fragility fracture in the underweight and obese groups was compared with the normal weight group via multiple logistic regression analysis. In model 1, which was not corrected, the odds ratio for fracture in the underweight group was 5.48 (95% CI 1.66–17.76) and 2.72 (95% CI 1.38–5.37) in the obese group. In model 2, which was corrected for age and family history and treatment history of osteoporosis, odds ratio for the underweight group was 5.14 (95% CI 1.77–14.93) and 3.12 (95% CI 1.50–6.48) in the obese group. In model 3, which was corrected for age, family history and treatment history of osteoporosis, history of rheumatoid arthritis, smoking status, drinking status, and moderate exercise, the odds ratio for the underweight group was 5.48 (95% CI 1.80–16.73) and 3.33 (95% CI 1.61–6.87) for the obese group (Table 2).

**Table 2:** Odds ratio (OR) of fragility fracture for postmenopausal women by multivariate logistic regression analysis

	OR (95% CI)
Normal weight (BMI ≥ 18.5 and < 25)	1 (Reference)
Underweight (BMI < 18.5)	
Model 1	5.48 (1.66–17.76)
Model 2	5.14 (1.77–14.93)
Model 3	5.48 (1.80–16.73)
Obesity (BMI ≥ 25)	
Model 1	2.72 (1.38–5.37)
Model 2	3.12 (1.50–6.48)
Model 3	3.33 (1.61–6.87)

Model 1: Crude

Model 2: Adjusted for age, family history of OP and treatment of OP

Model 3: Adjusted for age, family history of OP, treatment of OP, presence of rheumatoid arthritis, smoking status, alcohol consumption and moderate exercise

*BMI* body mass index, *OP* osteoporosis

Fragility fractures were classified as vertebral and non-vertebral fractures, and logistic regression analysis was performed after correcting for age, family history and treatment history of osteoporosis, history of rheumatoid arthritis, smoking status, drinking status, and moderate exercise. In the underweight group, the odds ratio for vertebral fracture was 5.49 (95% CI 1.31–23.09) times higher than that of the normal weight group. In the obese group, there was no statistically significant difference in the odds ratio for vertebral fracture, while the non-vertebral fracture odds ratio was observed to be 3.87 (95% CI 1.45–10.33) times higher. When non-vertebral fractures were compared after dividing them into hip and wrist, the obese group had an odds ratio for hip fracture 22.05 (95% CI 1.33–365.31) times higher and 3.85 (95% CI 1.35–10.93) times higher for wrist fracture (Table 3).

**Table 3:** Odds ratio (OR) of vertebral fracture (VF) and non-vertebral fracture (NVF) according to BMI

	VF	NVF		
	OR (95% CI)	OR (95% CI)		
	Total	Total	Hip	Wrist
Underweight (BMI < 18.5)	5.49 (1.31–23.09)	1.83 (0.22–15.60)	10.65 (0.38–295.97)	2.02 (0.24–17.29)
Normal (BMI ≥ 18.5 and < 25)	1 (Reference)	1 (Reference)	1 (Reference)	1 (Reference)
Obesity (BMI ≥ 25)	1.89 (0.59–6.00)	3.87 (1.45–10.33)	22.05 (1.33–365.31)	3.85 (1.35–10.93)

Adjusting for age, family history of osteoporosis, treatment of osteoporosis, presence of rheumatoid arthritis, smoking status, alcohol consumption and moderate exercise

*BMI* body mass index

## Discussion

The purpose of this study was to investigate the relationship between BMI and fragility fracture(s) in postmenopausal Korean women. In both the underweight and obese groups, the fragility fracture rates were 5.48 and 3.33 times higher, respectively, compared with the normal weight group. In particular, the underweight group exhibited an odds ratio for vertebral fracture that was 5.49 times higher, and the obese group exhibited an odds ratio for non-vertebral fracture that was 3.87 times higher. As such, the most significant finding of the present study was that postmenopausal women who were underweight or obese exhibited higher rates of fragility fracture(s).

The findings of high fragility fracture rate in the underweight group were consistent with previous studies; however, there were slight differences in fracture sites for each study. According to one study, underweight individuals with a BMI < 20 kg/m^2^ demonstrated a hip fracture rate twice as high as those with a BMI of 25 kg/m^2^ [18]. Another study found that for individuals with a BMI < 20 kg/m^2^, the non-vertebral fracture rate was 2.45 times higher [19]. The reason for this is that the risk for falls increases with lower BMI [20]. However, previous studies have shown that lower weight increases lumbar spine fractures, similar to the results of the present investigation. One study found that the decrease in BMI increased the vertebral fracture rate [21]. Other studies reported that lumbar spine fracture rate was 2.79 times higher for underweight individuals with BMI < 18.5 kg/m^2^ compared to those with BMI > 24 kg/m^2^ [22]. Studies have reported that the reason for high fragility fracture rate in underweight individuals is related to muscle loss. According to one study, sarcopenia is associated with older age, low BMI, and low bone density [23]. Although this explains the increase in fragility fracture rate [24], items for diagnosing sarcopenia were omitted in this study; as such, an accurate comparison could not be made.

In this study, there was no statistical difference in the rate of lumbar spine fractures in the obese group, which was similar to other previous studies. One study that examined changes in lumbar spine bone density in individuals with weight changes showed that individuals with weight gain had increased lumbar spine bone density compared to those with weight loss [25]. As a result, we can predict that as weight increases, spinal bone density increases, and the fracture rate decreases. Another study showed that high BMI and waist circumference in women were associated with a decrease in bone density due to increase in fat mass; however, whether these factors affect the lumbar spine fracture rate needs further study [26].

Recently, it was reported that obesity is related to the site of fragility fractures. One study found that obesity led to lower risk of lumbar and femur fractures [18]; however, another study showed that obesity increased the non-vertebral fracture rate. In particular, the proximal humerus fracture rate was 1.28 times higher, the femur fracture rate was 1.7 times higher, and the ankle fracture rate was 1.5 times higher [27, 28]. Another study reported that high BMI led to a decrease in the relative risk for fragility fracture in the spine, hip, and wrist, but an increase in fragility fractures of the ankle [29]. In postmenopausal women, obesity led to an increase in humerus fracture rates [30]. As such, one reason why obesity is associated with fractures could be that high body fat levels can reduce bone density. One study reported that the bone loss effect in the entire hip and the femoral neck was more prominent in Korean middle-age women with normal weight and a body fat percentage ≥ 36% [31].

The non-vertebral fracture rate was higher in the obese group, unlike the underweight group, which could be explained by the fact that the obese group exhibited significantly lower weight-corrected appendicular skeletal muscle mass compared with the normal weight group. Appendicular skeletal muscle mass is used as one metric to define sarcopenia [32], and it is common to use height-corrected appendicular skeletal muscle mass. However, the reason for comparing weight-corrected appendicular skeletal muscle mass in this study was based on a study in which Korean women with height-corrected appendicular skeletal muscle mass demonstrated a poor correlation with age-related muscle mass reduction; therefore, it was necessary to use weight-corrected appendicular skeletal muscle mass [33]. According to one study, sarcopenia with reduced appendicular skeletal muscle mass was associated with a decrease in bone density [34]. Although this decrease in bone density may be directly related to fractures, it is also possible that the risk for falls is increased due to a decrease in appendicular skeletal muscle mass [35].

Generalizing the results of the present investigation would be difficult given some following points. First, it was a cross-sectional study limited to postmenopausal women. Second, it was unclear when the BMD test performed and when the fracture occurred. Third, the BMI might have been calculated relatively high due to a decrease in height if there were a single or multiple vertebral fractures in the past. Further studies are required to consider the association between fragility fractures and other obesity indices such as waist circumference or waist to hip ratio as well as BMI. Fourth, the overweight group was inevitably included in the normal weight group for analysis because accurate analysis was difficult due to data limitations. Fifth, the percentage of underweight was only 2% of the total study population. As a relatively small number of people were classified as underweight, the general bone density characteristics of the group did not appear, and it is thought that the individual subjects' bone density characteristics were more reflected. This should be studied further through future researches.

## Conclusions

In conclusion, despite the various limitations, the most significant finding of this study was that underweight and obesity increased the rate of fragility fractures in postmenopausal women. In the end, there are various factors that affect fragility fractures, so further research will be needed, but the this study showed the impact of the body mass index.

## Data Availability

The original datasets used and analyzed during the current study are publicly available for academic purposes in the KNHANES website (http://knhanes.cdc.go.kr or https://knhanes.cdc.go.kr/knhanes/eng/index.do).

## Abbreviations

BMI: Body mass index
WHO: World health organization
